# Placenta-Enriched LincRNAs *MIR503HG* and *LINC00629* Decrease Migration and Invasion Potential of JEG-3 Cell Line

**DOI:** 10.1371/journal.pone.0151560

**Published:** 2016-03-29

**Authors:** Bruna Rodrigues Muys, Júlio Cesar Cetrulo Lorenzi, Dalila Luciola Zanette, Rafaela de Barros Lima e Bueno, Luíza Ferreira de Araújo, Anemari Ramos Dinarte-Santos, Cleidson Pádua Alves, Anelisa Ramão, Greice Andreotti de Molfetta, Daniel Onofre Vidal, Wilson Araújo Silva

**Affiliations:** 1 Department of Genetics, Ribeirão Preto Medical School, University of São Paulo, Ribeirão Preto, Brazil; 2 Center for Cell-Based Therapy (CEPID/FAPESP), National institute of Science and Technology in Stem Cell and Cell Therapy (INCTC/CNPq), Regional Blood Center of Ribeirão Preto, Riberão Preto, Brazil; 3 Center for Medical Genomics (HCFMRP/USP), Center for Integrative Systems Biology (CISBi–NAP/USP), Ribeirão Preto, Brazil; 4 Gonçalo Moniz Research Center, Oswaldo Cruz Foundation, Bahia, Brazil; 5 Molecular Oncology Research Center, Barretos Cancer Hospital, Barretos, Brazil; VU University Medical Center, NETHERLANDS

## Abstract

*LINC00629* and *MIR503HG* are long intergenic non-coding RNAs (lincRNAs) mapped on chromosome X (Xq26), a region enriched for genes associated with human reproduction. Genes highly expressed in normal reproductive tissues and cancers (CT genes) are well known as potential tumor biomarkers. This study aimed to characterize the structure, expression, function and regulation mechanism of *MIR503HG* and *LINC00629* lincRNAs. According to our data, *MIR503HG* expression was almost exclusive to placenta and *LINC00629* was highly expressed in placenta and other reproductive tissues. Further analysis, using a cancer cell lines panel, showed that *MIR503HG and LINC00629* were expressed in 50% and 100% of the cancer cell lines, respectively. *MIR503HG* was expressed predominantly in the nucleus of JEG-3 choriocarcinoma cells. We observed a positively correlated expression between *MIR503HG* and *LINC00629*, and between the lincRNAs and neighboring miRNAs. Also, both *LINC00629* and *MIR503GH* could be negatively regulated by DNA methylation in an indirect way. Additionally, we identified new transcripts for *MIR503HG* and *LINC00629* that are relatively conserved when compared to other primates. Furthermore, we found that overexpression of *MIR503HG2* and the three-exon *LINC00629* new isoforms decreased invasion and migration potential of JEG-3 tumor cell line. In conclusion, our results suggest that lincRNAs *MIR503HG* and *LINC00629* impaired migration and invasion capacities in a choriocarcinoma *in vitro* model, indicating a potential role in human reproduction and tumorigenesis. Moreover, the *MIR503HG* expression pattern found here could indicate a putative new tumor biomarker.

## Introduction

There is evidence that the X chromosome contains more genes related to sex and reproduction than we would be expected by chance [1]. Specifically, it is known that region Xq26 harbors critical genes responsible for placental and normal fetal development, including *PLAC1* gene [2], which expression is restricted to placenta [3]. Interestingly, PLAC1 protein is present in a variety of tumor types, and its expression was previously associated with the malignant phenotype [4–5]. Until recently, *PLAC1* has been the only candidate gene, within the chromosome region Xq26, relevant for normal placental development. Nevertheless, according to the most updated version of the human genome [2], new genes with unknown functions have been identified in this region, such as *PHF6*, *MIR503HG*, and *LINC00629*, the last two were previously known as *MGC16121 and CR596471*, *respectively*.

*MIR503HG* and *LINC00629* genes are mapped between *HPRT1* and *PLAC1* loci (Genome Browse–UCSC; Feb. 2009, CRCh37/hg19) in opposite orientations and are about 3kb apart from each other (S1 Fig). Both genes present three exons and CpG islands in their putative promoter regions. Also, they were described as long intergenic non-coding RNAs (lincRNAs): RNAs longer than 200 bp, non-translated and located between protein-coding genes. LincRNAs are involved in regulation of transcription, processing, and post-transcription pathways [6] and when deregulated, they are associated with several types of cancer [7].

Considering the significance of Xq26 region in embryo development and similarity between germinal and placental cells with tumors [8], we sought to characterize the structure, expression pattern, function, and regulation mechanism of *MIR503HG* and *LINC00629* genes.

## Results

### *MIR503HG* and *LINC00629* are highly expressed in reproductive tissues

**Fig 1:**
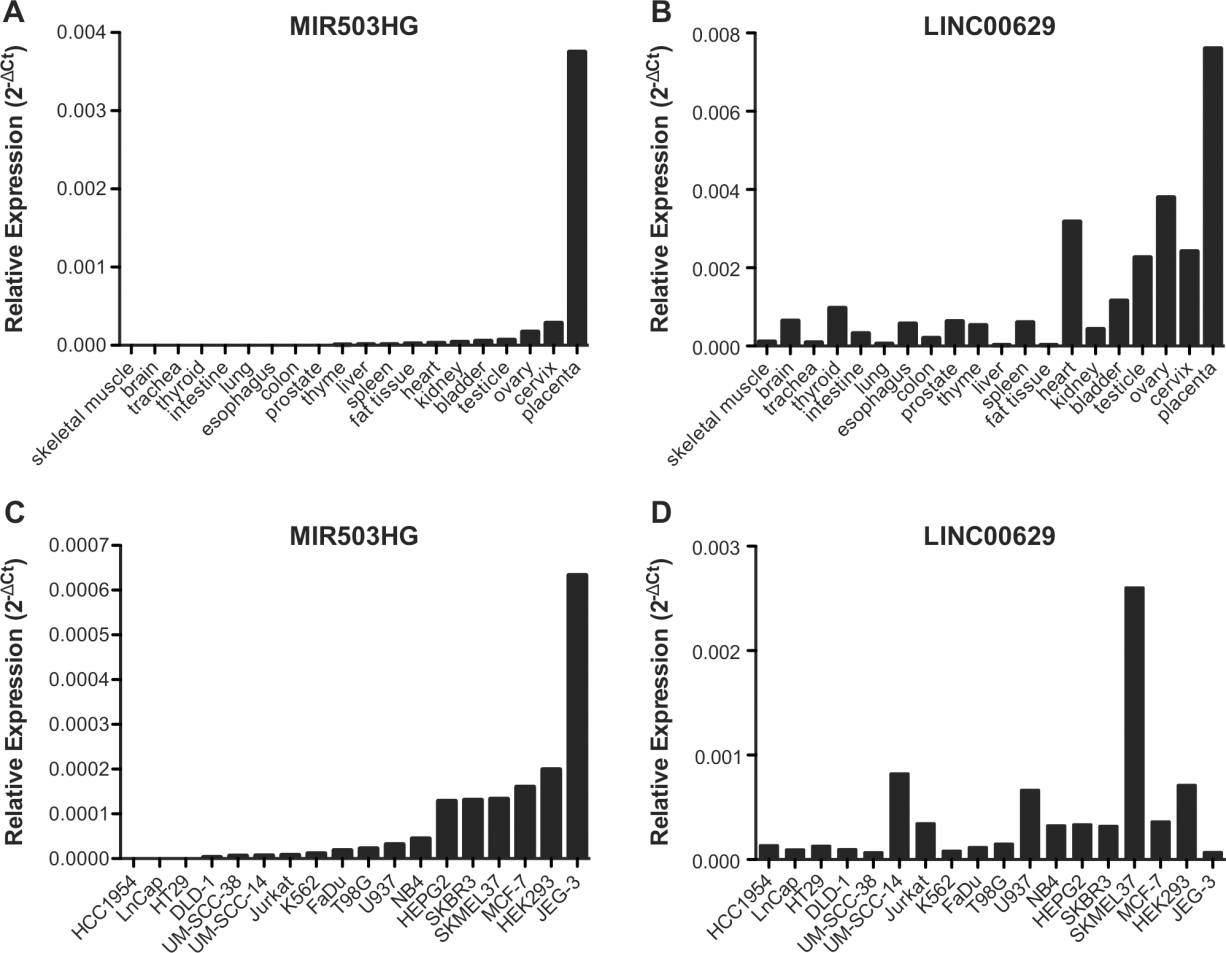
*MIR503GH* and *LINC00629* are highly expressed in placenta and also expressed in other reproductive tissues. A and B. *MIR503GH* and *LINC00629* expressions were determined using RNA samples from a commercial normal human tissue panel, and C and D. Expressions were determined using RNA samples from tumor cell lines. RT-qPCR evaluated the RNA expressions and further normalized by geometric mean from the endogenous genes GAPDH and HPRT.

*MIR503HG* and *LINC00629* genes are located in the same region as *PLAC1*, whose expression is restricted to placenta and recently was found to be expressed in cancer cells [2]. Therefore, we sought to determine the expression levels of *MIR503HG* and *LINC00629* in RNA samples from a commercial normal human tissue panel. We found that *MIR503HG* expression is almost restricted to the placenta (Fig 1A) and that *LINC00629* was also highly expressed in placenta and other reproductive tissues (Fig 1B). RT-qPCR analysis of 18 cancer cell lines revealed that *MIR503HG* is expressed in 50% (9/18) and *LINC00629* in 100% of them, considering as not expressed samples with Cts values above 34 (Fig 1C and 1D, respectively).

**Fig 2:**
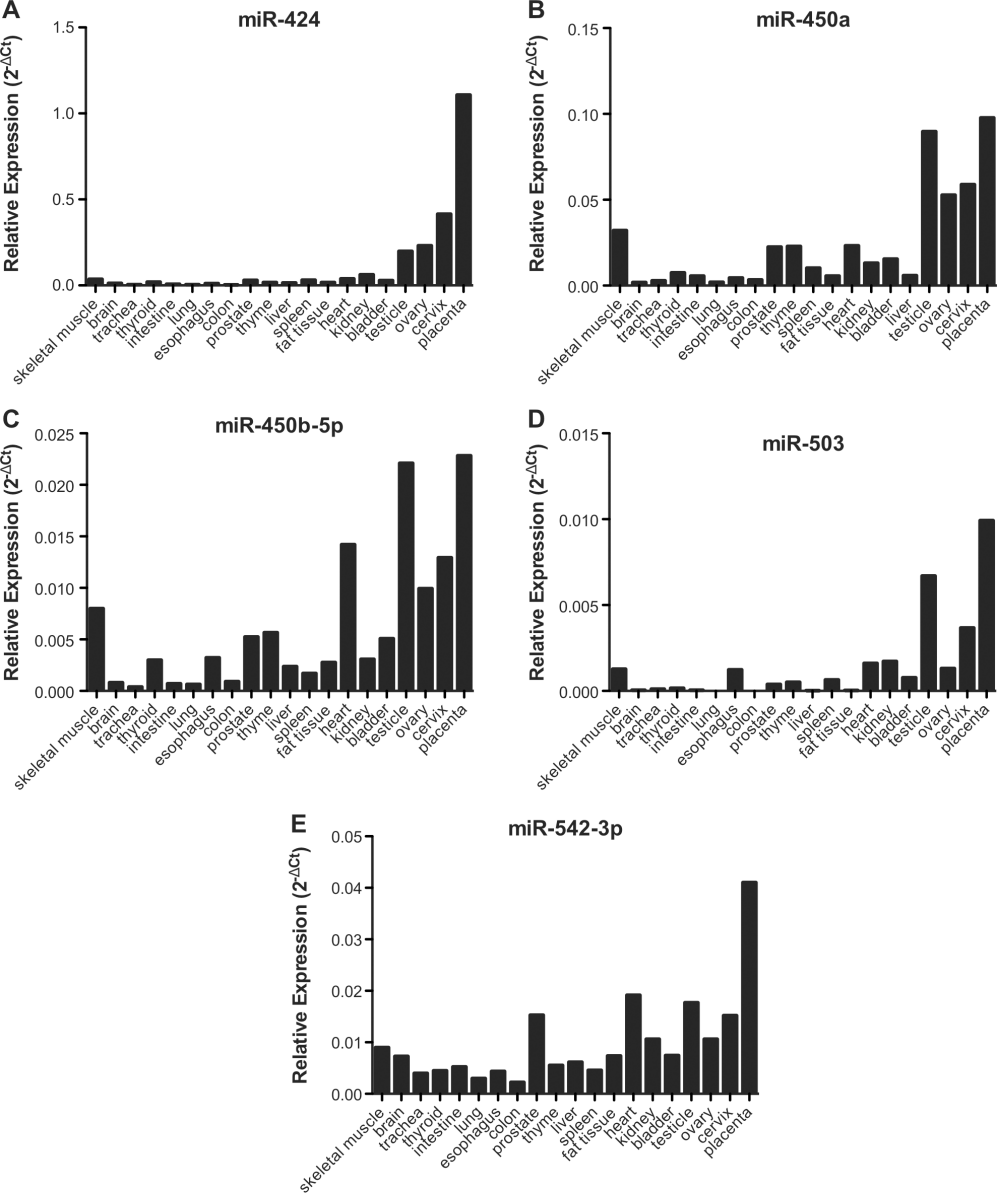
miRNAs flanking *MIR503HG* and *LINC00629* genes are in general more expressed in reproductive tissues, a similar expression pattern as the lncRNAs. A-E: RT-qPCR analysis using RNA samples from a commercial normal human tissue panel and further normalized by geometric mean from the snoRNAs *RNU24* and *RNU48*.

**Fig 3:**
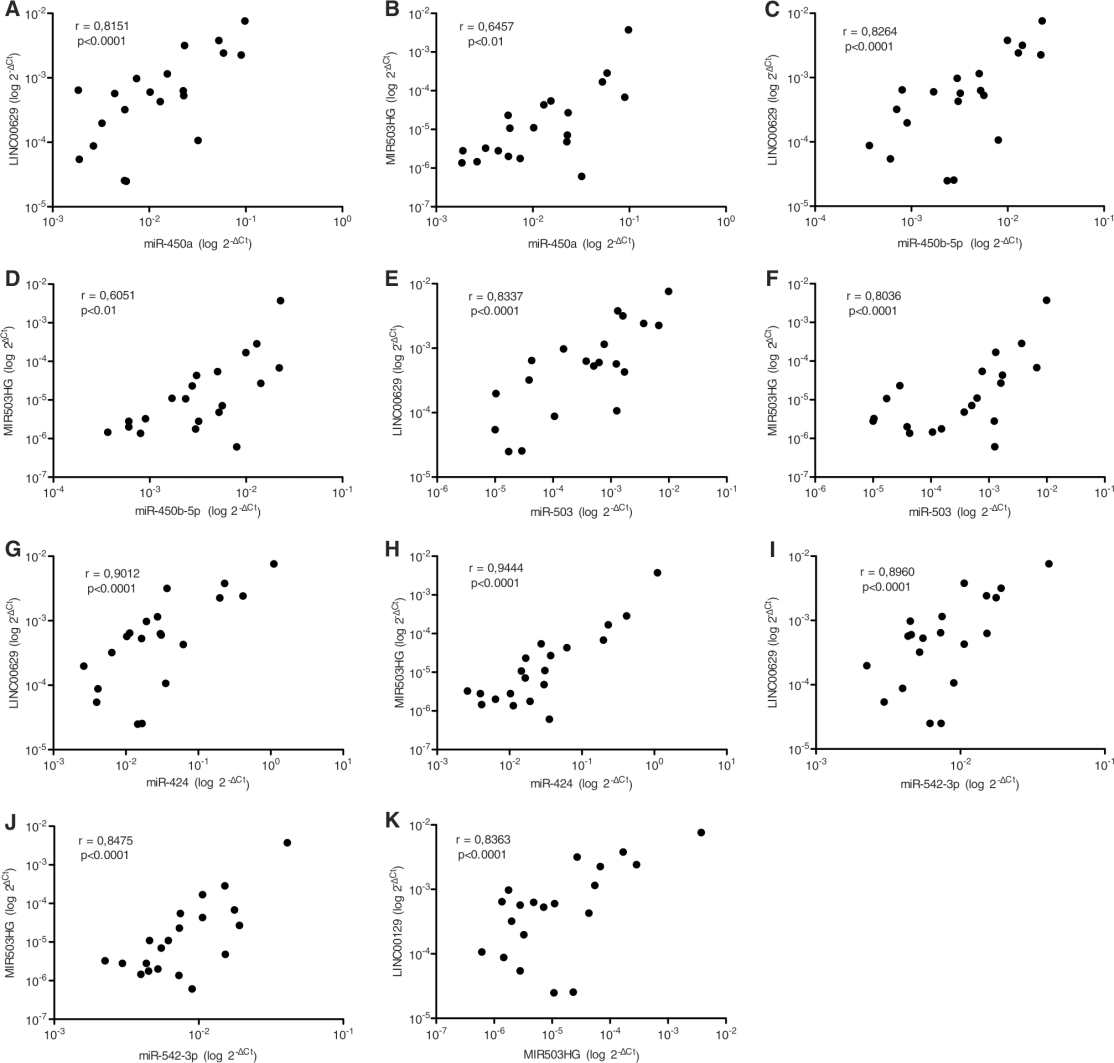
*MIR503HG* and *LINC00629* lncRNAs expression are related to each other and to their neighboring microRNAs, in normal tissue panel. A-J. Pearson Correlation (r) test between one of the lncRNAs and a neighbor microRNA.K. Pearson Correlation (r) test between *MIR503HG* and *LINC00629* genes.

MiRNAs flanking the same region also tended to be higher expressed in reproductive tissues as ovary, cervix, and placenta, similarly to *LINC00629* (Fig 2). Furthermore, using the normal tissue panel, we observed a significant positive correlation between *MIR503HG* and *LINC00629* lincRNAs (Fig 3K), and also between both lincRNAs and the neighboring miRNAs: miR-424, miR450a, miR-450b-5p, miR-542-3p and miR-503 (Fig 3A–3J).

### *MIR503HG* and *LINC00629* expression can be indirectly regulated by methylation

The presence of CpG islands in the promoter regions of both lincRNAs suggests that DNA methylation could regulate them. To test this possibility, we treated cancer cell lines with low expression of *MIR503HG* and *LINC00629* with the demethylating agent 5-Aza-2-deoxycytidine (5-Aza-dC).

**Fig 4:**
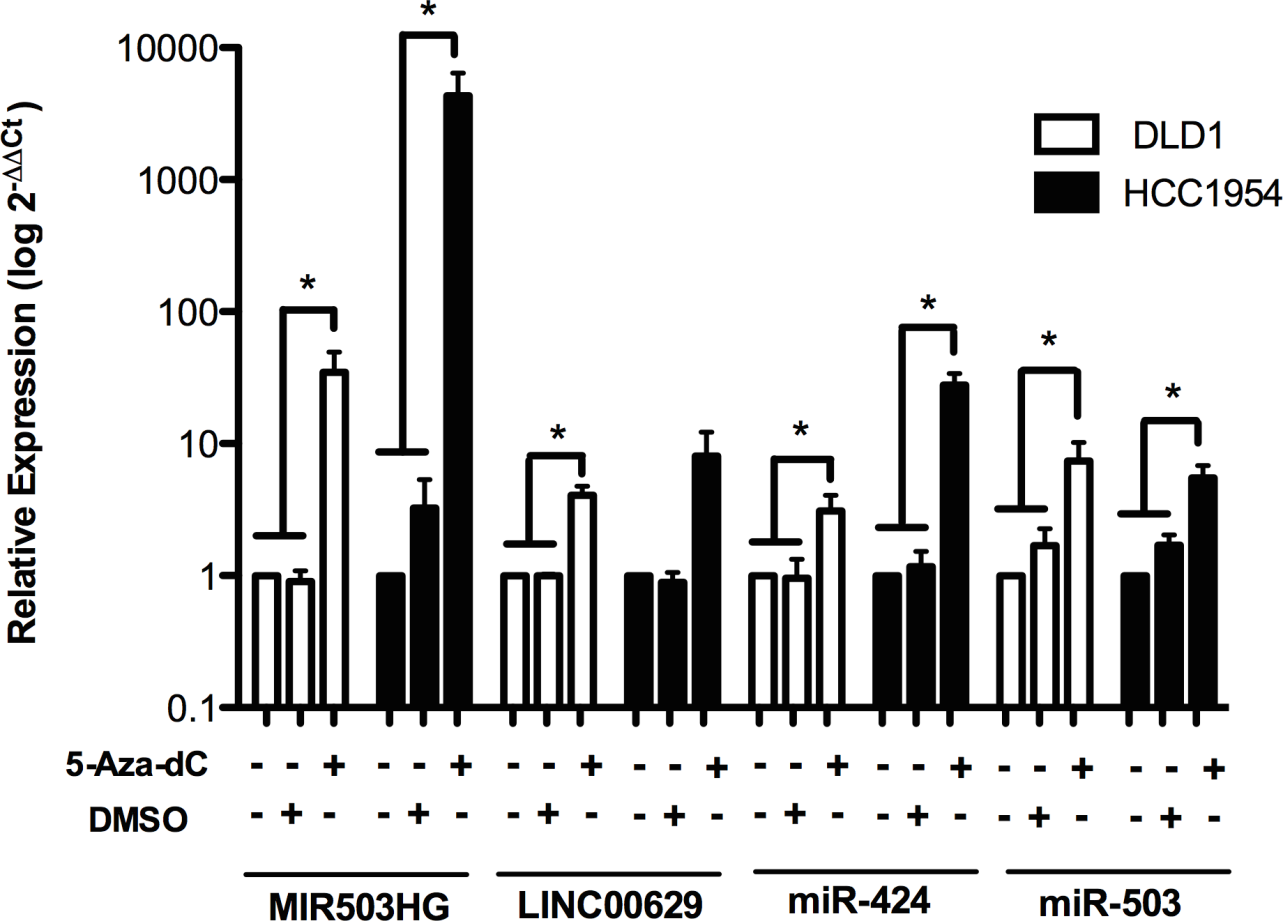
*MIR503HG* and *LINC00269* expression can be regulated by methylation. Expression levels of *MIR503HG*, *LINC00269*,and their neighbouring miRNAs—miR-424 and miR-503, were determined in RNA samples from breast and colon adenocarcinoma cell lines treated with 5-aza-2-deoxycytidine (5-Aza-dC) by RT-qPCR normalized to the geometric mean from *GAPDH* and *HPRT* genes (for *MIR503HG* and *LINC0026* genes) or snoRNAs *RNU24* and *RNU48* (for miRNAs). Relative expression was obtained from three independent experiments using samples without treatment as reference samples (2^-ΔΔCT^). *p <0.05 (*t*-test).

Treatment with 5-Aza-dC at 5 μM significantly increased *MIR503HG* expression in mammary gland tumor cell line (HCC1954) and the colorectal adenocarcinoma cell line (DLD-1). On the other hand, *LINC00629* expression was increased by treatment only in the DLD-1 cell line (Fig 4). Likewise, the miRNAs miR-424 and miR-503, which are mapped in the *MIR503HG* gene, also presented increased expression after 5-Aza-dC treatment (Fig 4).

**Fig 5:**
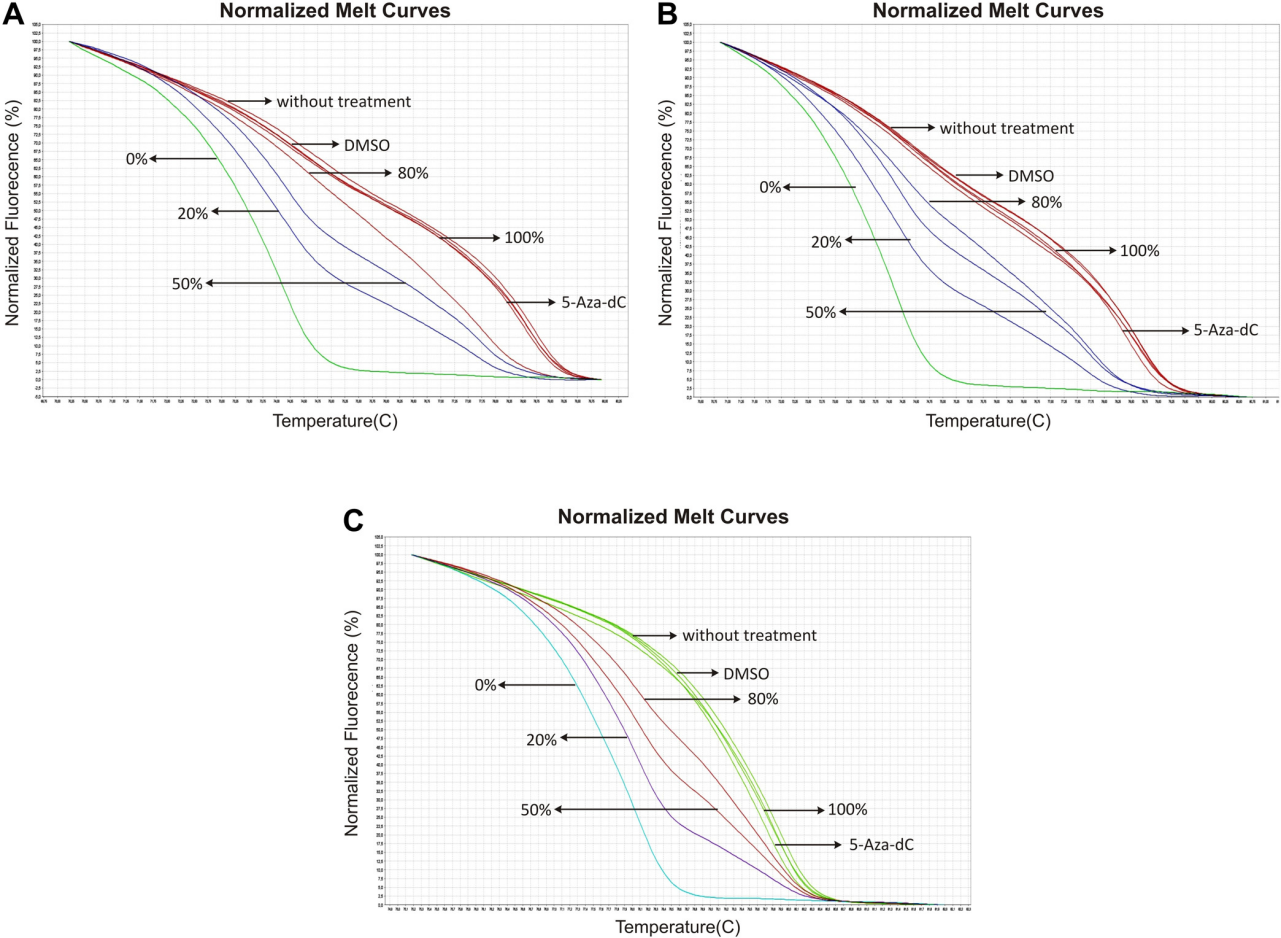
5-Aza-2-deoxycytidine (5-Aza-dC) treatment does not affect methylation status of the CpG islands near to putative promoter region of both genes. Representative normalized melt curves from samples treated with demethylating agent 5-Aza-dC. A-B. DNA analyzed from the CpG island at the promoter region of *MIR503HG* gene in DLD1 (a) and HCC1954 (b) cell lines. C. DNA analyzed from the CpG island at the promoter region of *LINC00629* gene in DLD1 cell line. Arrows indicate curves that correspond the percentage of methylation from reference, treatment, and control DNA samples. Images were obtained from High-ReSolution Melt Software v2.

Interestingly, Methylation Sensitive High-Resolution Melting (MS-HRM) revealed that methylation status of the CpG islands near to putative promoter region of both genes did not change after treatment. Both control and treated samples were 100% methylated (Fig 5). To prove that DNA global demethylation was effective after 5-aza-dC treatment, we digested DNA derived from treated samples using *MspI* and *HpaII* restriction enzymes. *HpaII* is sensitive to DNA methylation within the CCGG region and an isoschizomer of *MspI*. Comparing DNA band intensities in agarose gel from samples digested with *MspI* or *HpaII*, we verified that 5-aza-treated samples presented a lower methylation level than untreated samples (S2 Fig).

### *MIR503HG* and *LINC00629* new isoforms

Although *MIR503HG* and *LINC00629* genes have been recently validated, we have found some isoforms not previously reported nor deposited at *NCBI RNA reference sequences collection* (RefSeq). The new sequences identified for both genes were submitted to the GenBank database under accession numbers KM886853 (*MIR503HG2*), KM886854 (*LINC00629A*), KM886855 (*LINC00629B*), KM886856 (*LINC00629C*) and KM886857 (*LINC00629D*).

**Fig 6:**
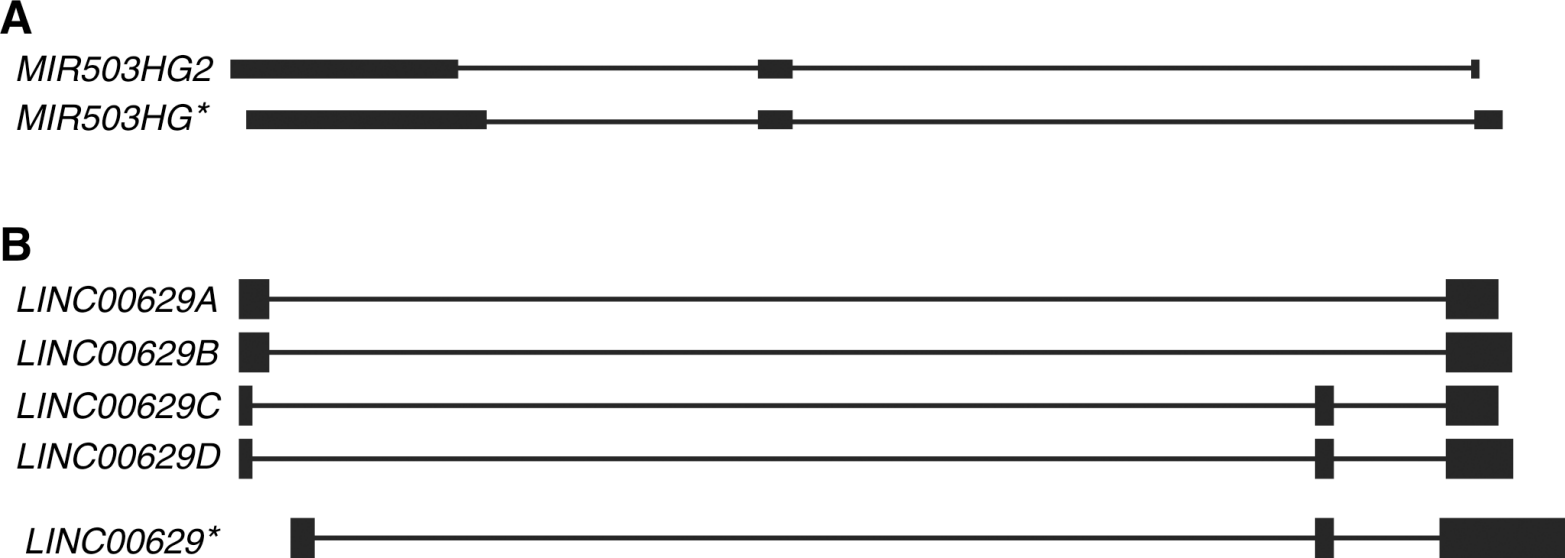
New isoforms of *MIR503HG* and *LINC00629* genes were identified. A. *MIR503HG* new isoform, named *MIR503HG*2. B. *LINC00629* four new isoforms containing either two exons (*LINC00629A* and *LINC00629B*) or three exons (*LINC00629C* and *LINC00629D*). The sequences were aligned by BLAT tool to the human genome (http://genome.ucsc.edu. *version*: *Feb*. *2009*, *CRCh37/hg19)*. *Reference isoforms.

Regarding to the *MIR503HG* gene, we identified an isoform that had a smaller 5’ region and a longer 3’ region when compared to the *MIR503HG* sequence, currently deposited in RefSeq (Fig 6A). We denominated this new isoform *MIR503HG2*. Likewise, *LINC00629* presented other four isoforms, two of them carried three exons and the other two had only two exons. In GenBank, we deposited the *LINC00629* isoforms: *LINC00629A* and *LINC00629B* (2 exons); *LINC00629C* and *LINC00629D* (3 exons). We observed that in the three-exon isoforms, the first exon was mapped in a different position than in the original sequence previous described at RefSeq. We found no similar transcripts to the isoforms containing two exons in the same data bank (Fig 6B). Isoforms with the same number of exons also differ by their length in poly A tail.

The expression profile for the two and three-exons *LINC00629* isoforms described herein is similar to normal tissues and cancer cell lines tested. However, the isoforms containing three exons were more expressed than the two-exons isoforms (S3 Fig).

### Conservation and secondary structure

**Fig 7:**
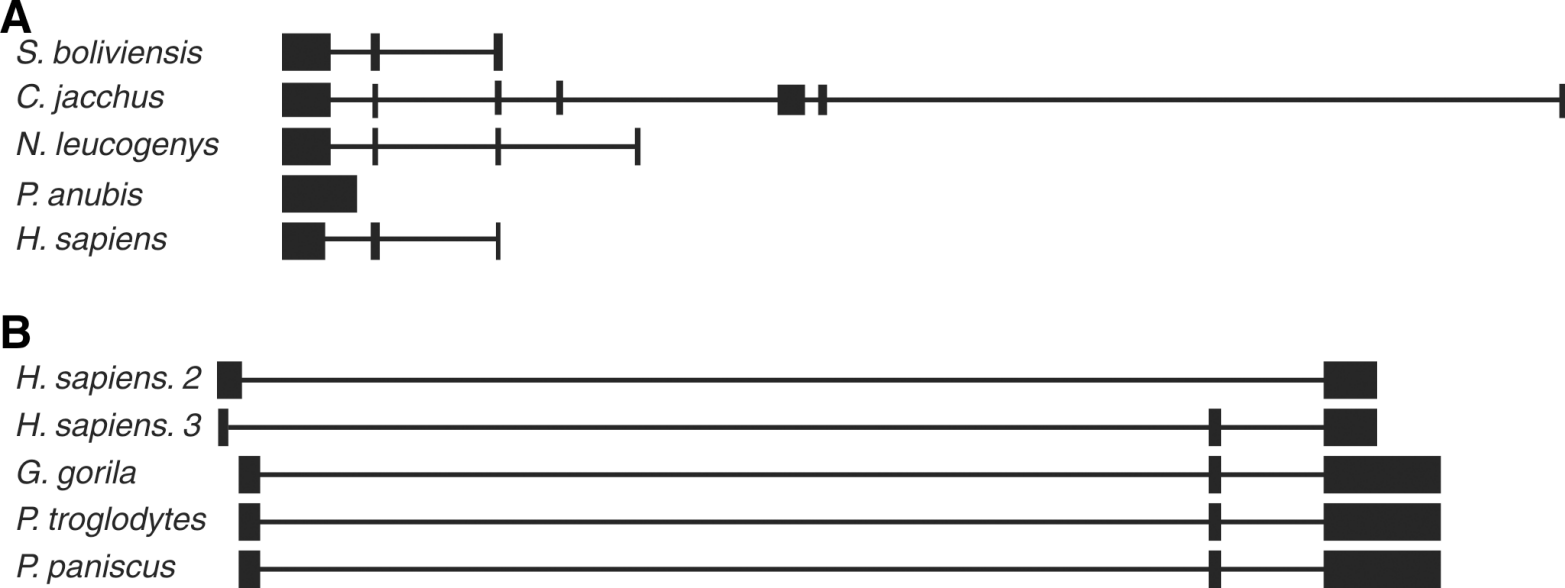
Evolutionary conservation of the new isoforms. A. Sequence obtained from *MIR503HG2* isoform (*H*. *sapiens*) aligned to human genome using BLAT tool (http://genome.ucsc.edu. *version*: *Feb*. *2009*, *CRCh37/hg19)* with similar sequences in *Saimiri boliviensis* (S. *boliviensis*), *Callithrix jacchus* (*C*. *jacchus*), *Nomascus leucogenys* (*N*. *leucogenys*) and *Papio anubis* (*P*. *anubis*) found in NCBI-BLAST. B. Sequences obtained from *LINC0026* isoforms comprising two exons (*H*. *sapiens*. 2) and three exons (*H*. *sapiens*. 3) aligned to human genome using BLAT tool (http://genome.ucsc.edu. *version*: *Feb*. *2009*, *CRCh37/hg19)* with similar sequences in *Gorilla gorilla* (*G*. *gorilla*), *Pan troglodytes* (*P*. *troglodytes*) and *Pan paniscus* (*P*. *paniscus*) found in NCBI-BLAST.

To evaluate the conservation and structure of the new isoforms, we compared them with similar sequences from other species deposited in the GenBank (RefSeq). The most similar sequences were found in other primates. Regarding *MIR503HG2*, the sequences with the highest identity were from *Nomascus leucogenys*, *Papio anubis*, *Saimiri boliviensis* and *Callithrix jacchus*. When aligned to the human genome, the last exons were apparently the most conserved (Fig 7A). For *LINC00629*, we only evaluated one of each two-exons or three-exons isoforms, once they were very similar, differing only in the 3’ end. For both types of isoforms, the most similar sequences were found in *Pan paniscus*, *Pan troglodytes* and *Gorilla gorilla* (Fig 7B).

Analysis of the secondary structure of *MIR503HG2* showed that the region corresponding to the exon 3, displayed a substructure similar to other primates (S4 Fig).

### *MIR503HG* is predominantly found in nucleus and *LINC00629* is evenly spread in JEG-3 choriocarcinoma cell line

**Fig 8:**
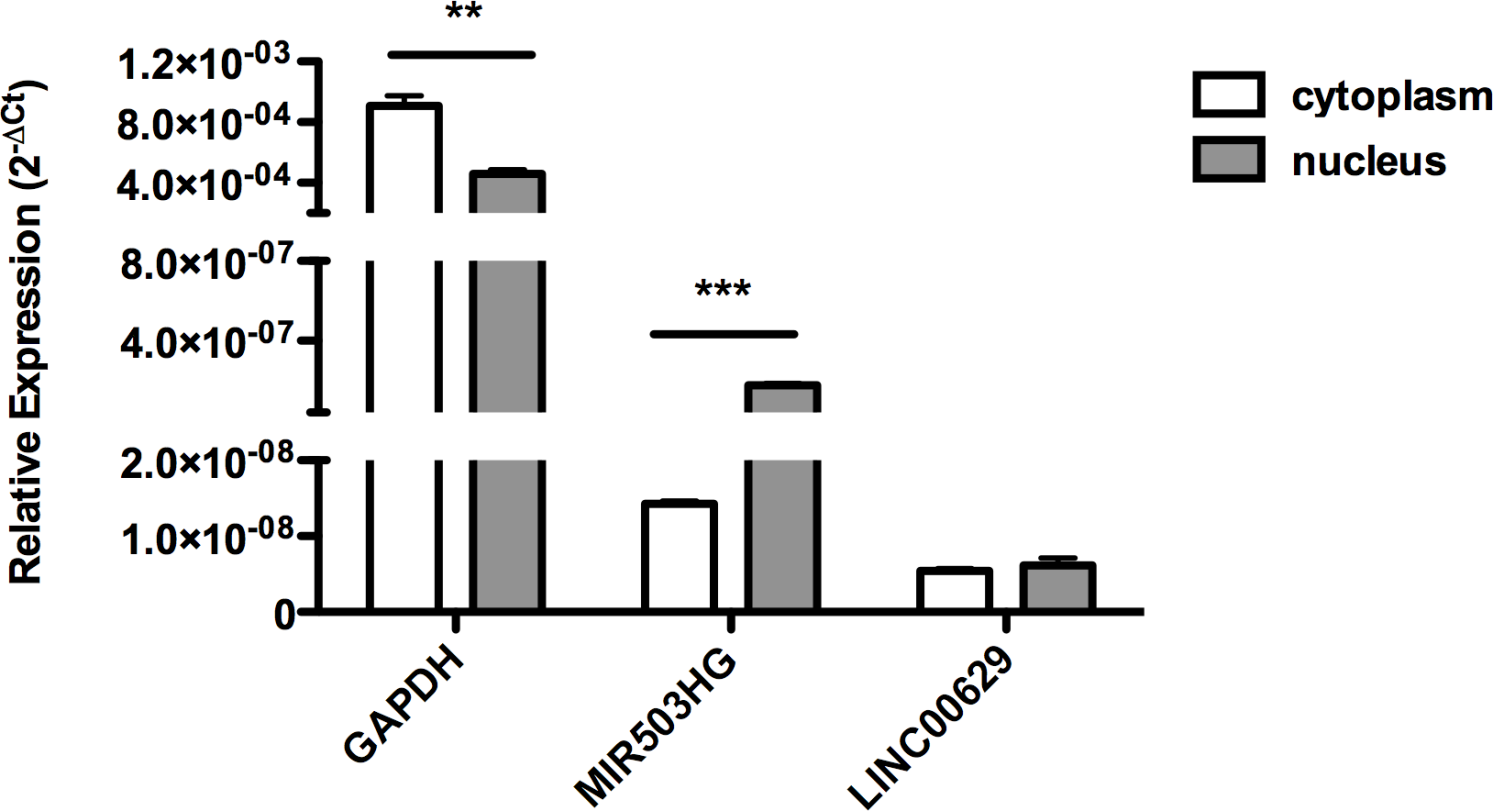
Cellular location of *MIR503HG* and *LINC00269* RNAs. RNA samples were extracted from nuclear and cytoplasmic fractions of JEG3 cells. RT-qPCR obtained relative expression from three experiments normalized to 18S ribosomal RNA. GAPDH fits as a control of predominantly cytoplasmic genes. **p <0.01 and ***p <0.001 (*t*-test).

To determine the cellular location from *MIR503HG* and *LINC00629* RNAs, we extracted RNA from nucleus and cytoplasm from JEG-3 cells cultured in normal conditions. Fig 8 shows that *MIR503HG* RNA was mainly found in the nucleus, about 12-fold higher expressed than in cytoplasm (p<0.001), and *LINC00629* was equally dispersed in cell compartments.

### *MIR503HG* and *LINC00629* inhibit migration and invasion in JEG-3 choriocarcinoma cell line

**Fig 9:**
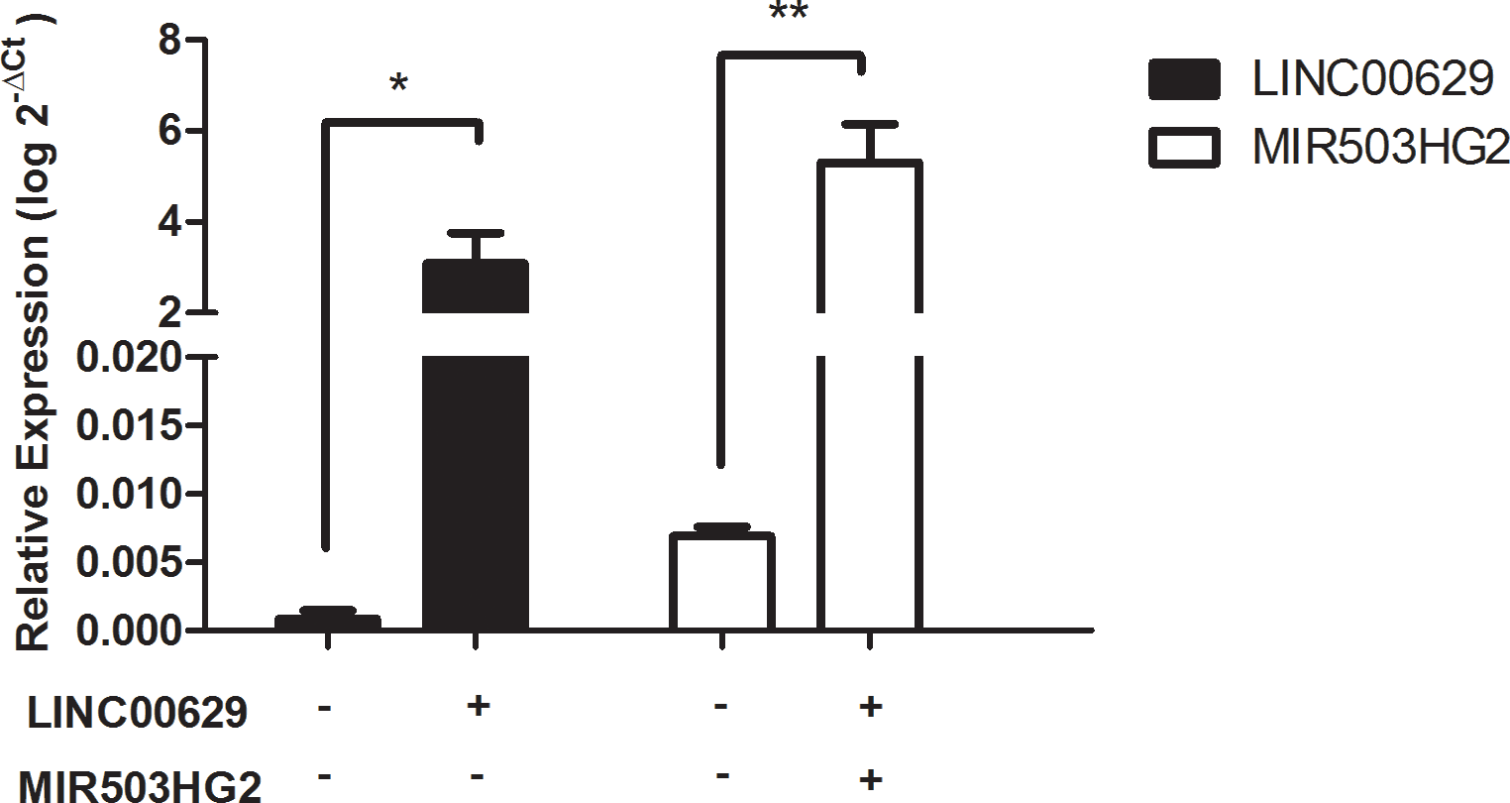
*MIR503HG2* or *LINC00269* were overexpressed in JEG-3 cell line. Plasmidial expression vectors containing *MIR503HG2* or *LINC00629* full-length sequences were transfected into JEG-3 cell line. Relative expression was analyzed by RT-qPCR normalized by the geometric mean from *GAPDH* and *HPRT* endogenous genes. The empty plasmid was used as a control. *p <0.05 and **p <0.01 (*t*-test).

Taking into account that *MIR503HG* and *LINC00629* were lower expressed in JEG-3 cell line than in normal placenta tissues (Fig 1), we used this cell line to overexpress the *MIR503HG2* and the 3-exon *LINC00629* isoforms (Fig 9).

**Fig 10:**
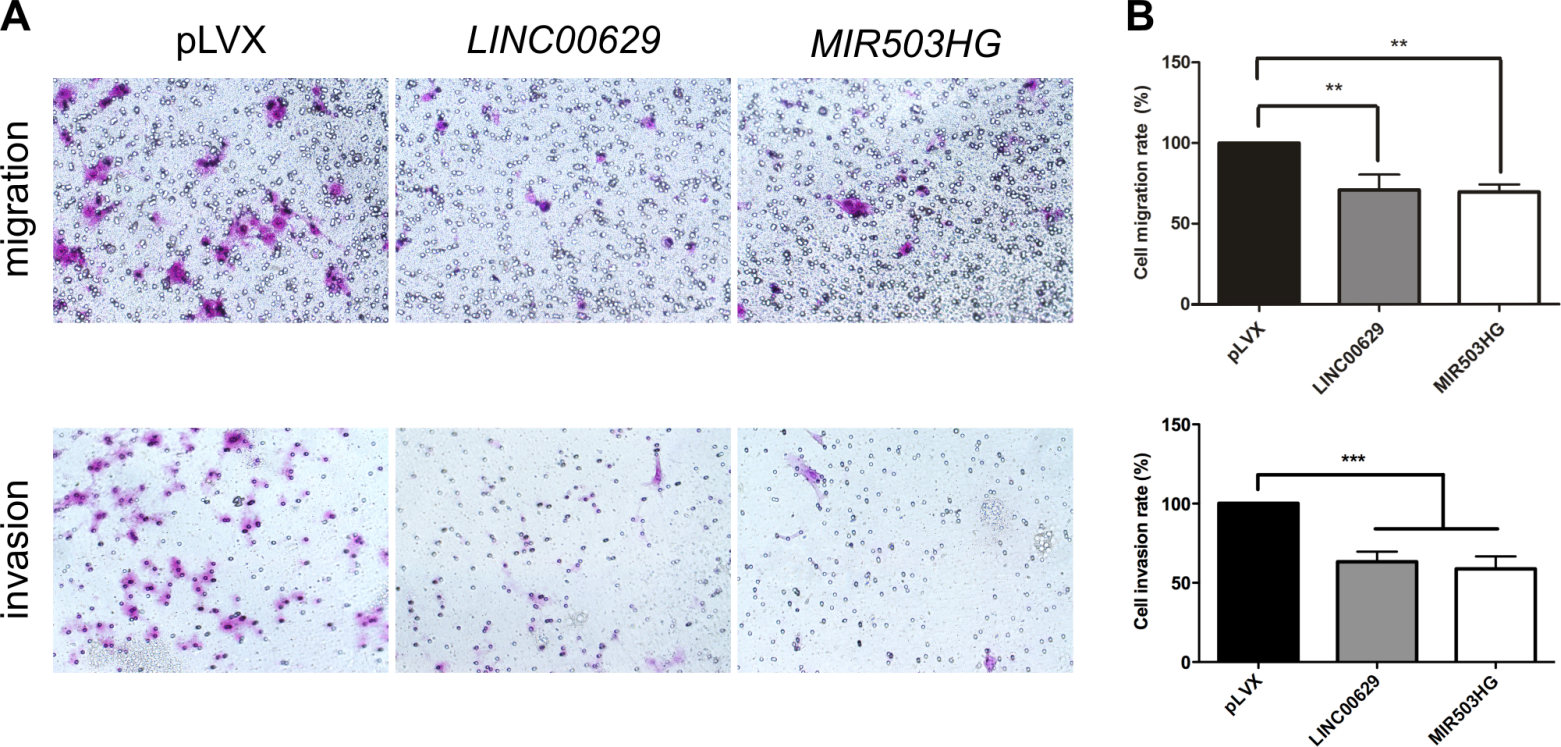
*LINC00629* or *MIR503HG2* overexpression decreased migration and invasion rates of JEG3 cell line. A. Representative figures from transwell migration (upper) and invasion assays (bottom) from cells containing empty (pLVX), *MIR503HG2* or *LINC00629* expression vectors. B. Graph bars are showing migration and invasion rates after 48 hours from transfection. The data represent the average of 2 or 3 experiments performed in triplicate. **p <0.01 and ***p <0.001 (ANOVA followed by Bonferroni post-test).

We found that the overexpression of both lincRNAs reduced the percentage of migrating cells around 30% (p<0.01) and invading cells in approximately 40% (p<0.001) (Fig 10A and 10B). However, there were no change of cells in S phase in cell cycle assay (S5 Fig), suggesting that cell proliferation not is affected by these lincRNAs.

## Discussion

Despite their functions, placenta, and germinative cells display some characteristics similar to tumor cells [8–9]. These include proliferation, migration, and invasion [10]. Consequently, the study of genes expressed in placenta and germinative cells could be considered a model system to investigate tumorigenic mechanism [10].

We showed here the characterization of *MIR503HG* and *LINC00629* genes. Both genes are described as lincRNAs located in Xq26 region, which contains other genes related to reproduction, fetal and placental development [2–3]. To our knowledge, gene expression profiles of both *loci* in normal and tumor cell lines have not been shown in the literature. According to Sasaki et al. [11] and Cabili et al. [12], most of the lncRNAs are expressed in a tissue-specific manner and probably regulate specific biologic process in each tissue.

*MIR503HG* and *LINC00629* genes were higher expressed in placenta compared to other normal tissues. *LINC00629* gene was also expressed in other tissues related to reproduction, such as cervix, ovary and testis. The same expression pattern was observed for miRNAs located near the studied *loci* (miR-424, miR-450a, miR-450b-5p, miR-503 and miR-542-3p). Takada et al. [13] showed in mice that the expression of miR-503 was restricted to placenta and ovary, supporting the suggestion that Xq26 region is involved in human reproduction and development. In a similar way, C19MC, which is exclusively expressed in placenta, contains miRNAs that are aberrantly expressed in specific human malignancies [14].

The positively correlated expression of *MIR503HG* and *LINC00629* genes and neighbor miRNAs suggests that all of them might be regulated simultaneously and that genetic and epigenetic alterations found in tumors could disrupt this control. However, on the opposite of our finding, Fiedler et al. [15] verified that *MIR503HG* repression up-regulated miR-424 in Human Umbilical Vein Endothelial Cells (HUVECs). We suggest that this correlation may be tissue related.

*MIR503HG*, *LINC00629*, miR-424 and miR-503 were negatively regulated by DNA methylation after treatment with the demethylating agent 5-aza-2-deoxycytidine. A previous study had already demonstrated that methylation regulates miR-503 in non-small lung cancer cell lines [16]. Nevertheless, the CpG dinucleotides from CpG islands located near the *MIR503HG* and *LINC00629* 5’ regions apparently have no effect on their expression. In this way, they must be regulated by demethylation in an indirect route. As observed in CTAs (cancer/testis-associated genes), the selective expression in germinal tissues and tumor cells can be regulated by DNA methylation [17]. In this way, despite the fact that our study is about non-coding RNAs, both genes seem to be regulated by the same mechanism.

*MIR503HG* and *LINC00629 loci* predicted from Expressed Sequence Tags (ESTs), which are more prone to have mistakes, mostly in its ends [18]. The RACE approach revealed that *LINC00629* locus transcribes two isoforms with two exons. Besides, the results showed that the three-exons isoforms presented higher expression than the two-exons isoforms, suggesting an important role in processes regulated by *LINC00629* gene.

Even though long non-coding genes are less conserved than coding genes regarding nucleotide sequence, they present highly conserved secondary structures [19]. The transcripts herein described are relatively conserved when compared to RNA sequences found in other primates. As stated by Necsulea et al. [20], the *MIR503HG* gene was originated at least 370 Myr ago in the tetrapod ancestor and, curiously, its expression pattern changed from predominantly testicular in ancestors to placental in eutherians.

Interestingly, we found that the last exon of the *MIR503HG2* transcript has a secondary substructure, which is very similar to transcripts from *Nomascus leucogenys*, *Callithrix jacchus* and *Saimiri saimiri*. The presence of a domain that is evolutionarily conserved in nucleotide sequence and RNA secondary structure suggests that it may represent a functional domain [21], and probably could be the most important region of these transcripts.

Functions of lncRNAs depend on cellular location, and most lincRNAs are located in the nucleus [22], as we found *MIR503HG* in JEG-3 cells. This suggests that it could act organizing sub-structures, altering the chromatin state or regulating gene expression [23]. On the other hand, *LINC00629*, which is evenly spread in nucleus and cytoplasm, may have a different role in global cellular function.

Functional assays demonstrated that overexpression of *MIR503HG2* and *LINC00629* three-exon isoform (*LINC00629C* and *LINC00629D*) decreased cell migration and invasion rates, indicating a potential role in tumorigenesis. Besides, once both lincRNAs are enriched in placenta and located in Xq26 region, we suggest that they could act as tumor suppressors in choriocarcinoma, which lost its ability to cease invasion and migration. Nevertheless, Fiedler et al. [15] verified that *MIR503HG* suppression inhibited migration and proliferation in HUVEC cells, indicating that its action must be influenced by cell context or isoform type.

Here we have characterized the structure, regulation by methylation and function of *MIR503HG* and *LINC00629* genes. Based on their expression profile and their effects on migration and invasion in a model of choriocarcinoma, our study suggests a potential role for *MIR503HG* and *LINC00629* genes in tumorigenesis and human reproduction, considering the similarity among normal placenta and germinal tissues to tumors. Additionally, the expression pattern found for *MIR503HG* could indicate a putative new tumor biomarker.

## Materials and Methods

### Cell culture experiments

For this work we used commercial human cell lines. All procedures were performed after approval of the Internal Human Ethics Committee (13867/2011) at the Clinical Hospital of the Medical School of Ribeirao Preto (Brazil).

Mammary gland tumor cell line HCC1954 and colorectal adenocarcinoma cell line DLD-1 were purchased from the American Type Culture Collection (ATCC) and cultivated in RPMI medium (Gibco, Catalog No. 31800–022). The JEG-3 cell line, from human choriocarcinoma, as well as FaDu, UM-SCC-14 and UM-SCC-38 cell lines, head and neck squamous cell carcinoma, were kindly provided by Dr. Eloiza H. Tajara from the School of Pharmaceutical Sciences–UNESP. JEG-3 cells were cultured in Eagle's Minimum Essential Medium (ATCC, Catalog No. 30–2003). All mediums were supplemented with 10% FBS (GE Healthcare, Catalog No. SH30071.03) and 1% penicillin-streptomycin (Sigma-Aldrich Co., Catalog No. P4333). All other cell lines utilized were purchased from ATCC or DSMZ and cultured according to recommended mediums. Cells were kept at 37°C and 5% CO_2_.

### Plasmid construction

Plasmids expression vectors containing GFP and the *MIR503HG2* or *LINC00629* full-length sequence were constructed using pLVX-IRES-ZsGreen Vector (Clontech, Catalog No. 632187, modified for restriction enzymes positions), EcoRI and BamH1 restriction enzymes (New England BioLabs Catalog No. R0101S and R0136S, respectively). We used cDNA derived from placenta tissue from a panel of 20 normal tissues RNA (FirstChoice Human Total RNA Panel Survey, Ambion, Catalog No. AM6000) to generate the lincRNAs full sequences. We used the following pair of primers to amplify *MIR503HG2*: MIR F: 5’ GGATCCGCTCCCCGCGAGGCCGGCT 3’ and MIR R: 5’ GAATTCGGACAGTTGCCC ATATTAAC 3’, and LINC F 5’ GGATCCACTGGGCGCCCAGAGTAA 3’ and LINC R 5’ GAATTCGAGAGTGACTTG CAGTCTTGTG 3’ to amplify *LINC00629* three-exon isoform (*LINC00629C* or *LINC00629D*).

### Plasmid transfection

The lincRNAs in this study were transfected in JEG-3 cell line using Lipofectamine 2000 reagent (Thermo Scientific, Catalog No. 11668–019) together with 500 ng of vector DNA for each well in 24 well plates. As a negative control, we used the empty vector. Experiments were performed 48 hours after transfection. The transfection efficiency and cell viability were evaluated by GFP expression and PI staining, using FACS Calibur flow cytometer (Becton Dickinson).

### Migration and invasion assays

Cell migration was evaluated in 24-well transwell plates (Greiner BioOne, Catalog No 662638). Matrigel Invasion Chamber (Corning, Catalog No. 354480) replaced the filters, in the invasion assay. Cells from the upper compartment were removed with a cotton swab and cells that migrated to the lower face of the filter were fixed in 4% formaldehyde (in PBS) and stained with 0.5% crystal violet. The number of cells was manually counted using Image J software. All experiments were performed two to three times, independently.

### Cell cycle assay

Cell cycle assay was done synchronizing cells by FBS starving for 24 hours before vectors transfection. After 48 hours post transfection, cells were fixed with ice-cold absolute ethanol overnight, added to RNAse A and PI and analyzed in FACS Calibur flow cytometer. All experiments were performed three times, independently.

### 5-Aza-dC treatment

For 5-Aza-,2-deoxycytidine (Sigma-Aldrich Co., Catalog No. A3656) treatment, 1.3x10^5^ DLD-1, and HCC1954 cells were cultured in each well of a six-well plate. After 24 hours, 5-Aza-2-deoxycytidine diluted in DMSO (Sigma-Aldrich Co., Catalog No. D2650) was added to a final concentration of 5 μM. Mediums were changed each 24 hours adding fresh drug each day, for three days. At the end of the experiment, the cells were harvested, and the viability analyzed using trypan blue (Life, Catalog No. 15250–061). All experiments were performed three times, independently.

### DNA and RNA extraction

RNA and DNA from cells were extracted with Trizol Reagent (Life, Catalog No. 15596–018), according to the manufacturer’s protocol.

### RT-qPCR analysis

To evaluate the expression profile of the lincRNAs, we used RNA extracted from cancer cell lines and a panel of 20 normal tissues (FirstChoice Human Total RNA Panel Survey, Ambion, Catalog No. AM6000). Reverse transcription was performed with High Capacity cDNA Reverse Transcription Kit (Thermo Scientific, Catalog No. 4368813), according to supplier´s protocol.

The RT-qPCR analysis was performed using Taqman Gene Expression Assay (Applied Biosystems) or IDT (Integrated DNA Technologies) probes. For *MIR503HG* RefSeq isoform we used the assay Hs03681341_m1 (Applied Biosystems) and for both RefSeq and *MIR503HG2* isoforms we utilized Hs.PT.58.2631940 (Integrated DNA Technologies). For *MIR503HG2* specific isoform, we used a custom probe with the following sequences: primer F: 5`CAG CCT TCC TGA AAG ACC A 3`; primer R: 5`TGT TGA TGT AGT GTT CCT GGG T 3`and probe: 5`CT CCA GTG G A CGC CTG CAG G 3`(Integrated DNA Technologies). For *LINC00629* gene, we used the assay 186830593 (Applied Biosystems) for *LINC00629*A and *LINC00629*B isoforms and the assay Hs04274538_m1 (Applied Biosystems) for RefSeq, *LINC00629*C, and *LINC00629*D isoforms. Expression levels were normalized with endogenous genes *GAPDH* (Applied Biosystems, Catalog No. Hs02758991_g1) and *HPRT1* (Applied Biosystems, Catalog No. Hs02800695_m1). MiRNAs expression analyzes were performed using TaqMan miRNA Assay (Applied Biosystems), for miR-424 (Catalog No. 000604), miR-450a (Catalog No.002303), miR-450b-5p (Catalog No.002207), miR-542-3p (Catalog No.001284) and miR-503 (Catalog No.001048). The snoRNAs *RNU24* (Catalog No. 001001) and *RNU48* (Catalog No. 001006) were used as endogenous genes. All reactions were performed in duplicates at 7500 Fast Real-Time PCR System (Applied Biosystems, Catalog No. 4351107) with Taqman Universal PCR Master Mix (Applied Biosystems, Catalog No. 4369016), using 4μl from diluted cDNA in a final reaction volume of 10 μl. qPCR conditions were: 1 cycle of 10 min at 95°C, followed by 40 cycles of 94°C, 15s and 60°C for 1 min.

All the expression data, except the ones from the demethylating treatment, were analyzed by the formula 2^-ΔCt^, in which ΔCt value was calculated using the geometric mean from endogenous genes. For the demethylating treatment experiment, we used 2^-ΔΔCt^, with the same endogenous and considering samples without treatment as a reference sample.

### Nucleus and cytoplasm expression assay

For nucleus and cytoplasm RNA separated extraction, we used PARIS Kit (Thermo Scientific, Catalog No. AM1921) according to supplier’s protocol and utilized *GAPDH* RNA (Applied Biosystems, Catalog No. Hs02758991_g1), predominantly cytoplasmic, as a control. We normalized the expression levels with endogenous rRNA 18S (Applied Biosystems, Catalog No. 4319413E) and expression analysis was performed as the previous item.

### DNA methylation assay

The DNA obtained from cell lines were subjected to treatment with sodium bisulfite, which is based on deamination of unmethylated cytosines to uracil and maintenance of methylated cytosines, in the presence of NaOH and sodium bisulfate [24]. For this procedure, we used EpiTect Bisulfite Kit (Qiagen, Catalog No. 59104), according to manufacturer's instructions.

The Methylation Sensitive High-Resolution Melting (MS-HRM) method allows analyzing the methylation percentages among different converted bisulfite DNA samples. This method is based on different dissociation times from double strand to single strand DNA, among distinctive methylated samples after PCR [25].

We utilized standard DNA fully methylated and no-methylated from EpiTect Control DNA (Qiagen, Catalog No. 59655) diluted in different concentrations (0%, 20%, 50%, 80% and 100% methylated) that we used as a reference.

To perform the HRM-MS method were used 10 ng of modified DNA sample, MeltDoctor HRM Master Mix (Applied Biosystems, Catalog No. 4415440) 1X and 0.3 μM of each primer sequence (forward and reverse) to a final volume of 20 μL. The reactions were performed in replicate using 7500 Fast Real-Time PCR System (Applied Biosystems, Catalog No. 4351107). The following cycle was used: 95°C (10 minutes), followed by 40 cycles of 95°C (15 seconds) and 60°C (1 minute); succeeded by the corresponding melting phase at 95°C (10 seconds), 60°C (1 minute), 95°C (15 seconds) and 60°C (15 seconds).

For CpG island located near to the 5' *MIR503HG* gene end, we used the following pair of primers: F MG HRM-MS: 5' GTTTATGCGTTTTAGTTTAGTTAGG 3' and R MG HRM-MS: 5' CGTATTCCTACCACCAAATACC 3'. For CpG island located near to the 5' *LINC00629* gene end, it was analyzed by the following pair of primers: CR F HRM-MS: 5'CGGGGTGGGGATTTTTTG3' and R CR HRM-MS: 5'ACAACTACGACCTCCCTC3 '. For both pairs, 60°C was used as annealing temperature.

The DNA derived from three independent experiments were analyzed in High ReSolution Melt Software v2 (Applied Biosystems, Catalog No. 4397808).

### Rapid Amplification of cDNA Ends (RACE)

We used the GeneRacer Kit (Invitrogen, Catalog No. L1502-01) as indicated in the manufacturer’s protocol to the *MIR503HG* and *LINC00629* genes. For the definition of the 5’ and 3’ regions of *MIR503HG* gene we used the RNA from the cell lines HEK293 and MCF-7 and, for the *LINC00629* gene, we used the UM-SCC-14 cell line. The following primers were used: *MIR503HG* 5’ region Reverse primer: 5’ GGAGTACAGCCCACTGTTTT 3’, and *LINC00629* 5’ region Reverse primer: 5’ GCTGAATAACGGATTACCCC 3’. *MIR503HG* 3’ region Forward primer: 5’ GCCAGCCAGCCTTCCTGAAA 3’, and *LINC00629* 3’ region Forward primer: 5’ GGG GTAATCCGTTATTCAGC 3’.

The sequences obtained by RACE technique and by PCR regarding the CpG islands were evaluated using the software Codoncode Aligner (CodonCode Corp.) and aligned to the human genome using the BLAT tool [26] (website: http://genome.ucsc.edu/).

### Conservation and secondary structure analyses

Nucleotide sequences obtained by RACE technique were analyzed by The Basic Local Alignment Search Tool (NCBI-BLAST) [27–29], searching for similar RNA sequences contained in the NCBI Reference RNA sequences (RefSeq_rna) [28]. Then, sequences similar to nonhuman RNA were compared to the studied genes and further aligned to the human genome, through the BLAT tool [26] (website: http://genome.ucsc.edu/). Additionally, we predicted the common RNA structure using the TurboFold algorithm, available in RNAstructure platform (http://rna.urmc.rochester.edu/RNAstructureWeb/) [29], which presents the common RNA structures with the lowest free energy values.

### Statistics Analysis

The expression data derived from cancer cell lines and normal tissue were analyzed with Pearson’s correlation coefficient, using expression values found for each gene and the miRNA. For the demethylating treatment experiment, transfected cells assays and nucleus/cytoplasm expression assay, we used Student's t-test. For migration and invasion assays, we used ANOVA followed by Bonferroni post-test. All the statistical analysis were performed with GraphPad Prism 4 software and p<0.05 was considered as significant.

## Supporting Information

S1 Fig Schematic figure showing *MIR503HG*, *LINC00629* and neighboring miRNAs positions in Xq26 region. Lighter gray bars at the bottom represent CpG islands. Adapted from Genome Browse–UCSC; Feb. 2009, CRCh37/hg19.
(JPG)

S2 Fig Global DNA methylation assay of cell lines treated with 5-Aza-dC. Genomic DNA samples from cell lines HCC1954 and DLD-1 treated with vehicle (DMSO), or 5 μM 5-Aza-dC were digested with *MspI* or *HpaII* restriction enzymes and loaded in a one percent SYBR stained agarose gel. *HpaII* is sensitive to DNA methylation within the CCGG region and an isoschizomer of *MspI*. ND: non-digested DNA.
(TIF)

S3 Fig Expression of *LINC00629* isoforms. A. *LINC00629* isoforms expression pattern in normal tissue panel. B. *LINC00629* isoforms expression in cancer cell lines. The endogenous *GAPDH* and *HPRT* genes were used for normalization of samples.
(TIF)

S4 Fig Secondary common structures among the *MIR503HG2* isoform from human and the similar ones found in other species. The circulated areas represent the most similar substructure localized in the last exon. The secondary structure was obtained through RNAstructure (http://rna.urmc.rochester.edu/RNAstructureWeb/) using the algorithm TurboFold.
(TIF)

S5 Fig Percentage of cells in S phase after *MIR503HG2* or *LINC00269* overexpression in the JEG-3 cell line. After 48 hours from transfection, cells were fixed with ice-cold absolute ethanol overnight, added to RNAse A and PI and analyzed in FACS Calibur flow cytometer. pLVX: empty expression vector.
(TIFF)
