# Supplementary figures and images for: Placenta-Enriched LincRNAs MIR503HG and LINC00629 Decrease Migration and Invasion Potential of JEG-3 Cell Line
Source: PLoS One. 2016 Mar 29;11(3):e0151560. doi: 10.1371/journal.pone.0151560 (PMC4833476; doi:10.1371/journal.pone.0151560)

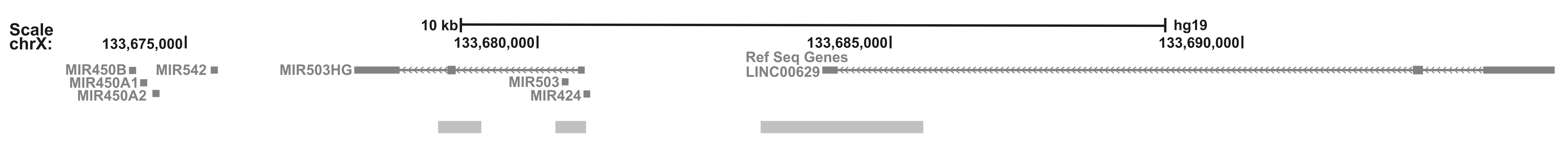

Supplement: S1 Fig — Lighter gray bars at the bottom represent CpG islands. Adapted from Genome Browse–UCSC; Feb. 2009, CRCh37/hg19. (JPG) [file pone.0151560.s001.jpg]

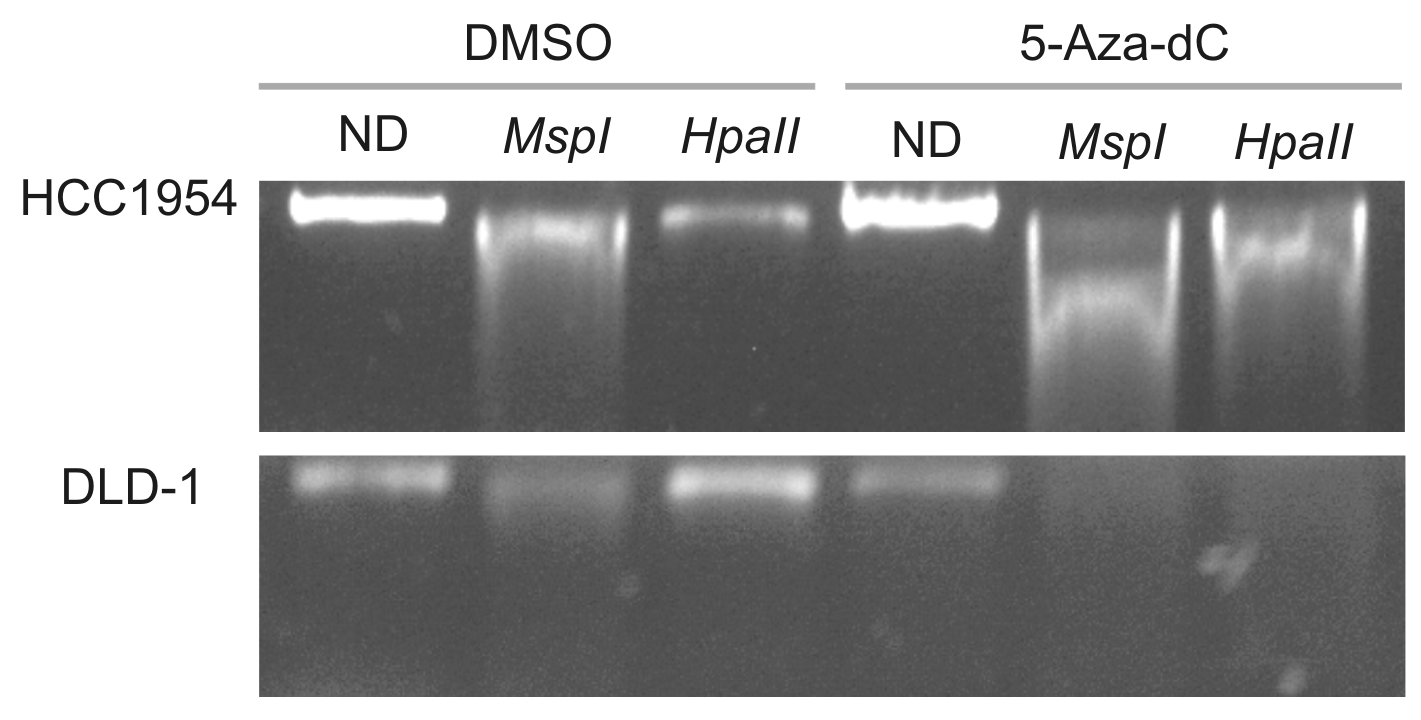

Supplement: S2 Fig — Genomic DNA samples from cell lines HCC1954 and DLD-1 treated with vehicle (DMSO), or 5 μM 5-Aza-dC were digested with MspI or HpaII restriction enzymes and loaded in a one percent SYBR stained agarose gel. HpaII is sensitive to DNA methylation within the CCGG region and an isoschizomer of MspI. ND: non-digested DNA. (TIF) [file pone.0151560.s002.tif]

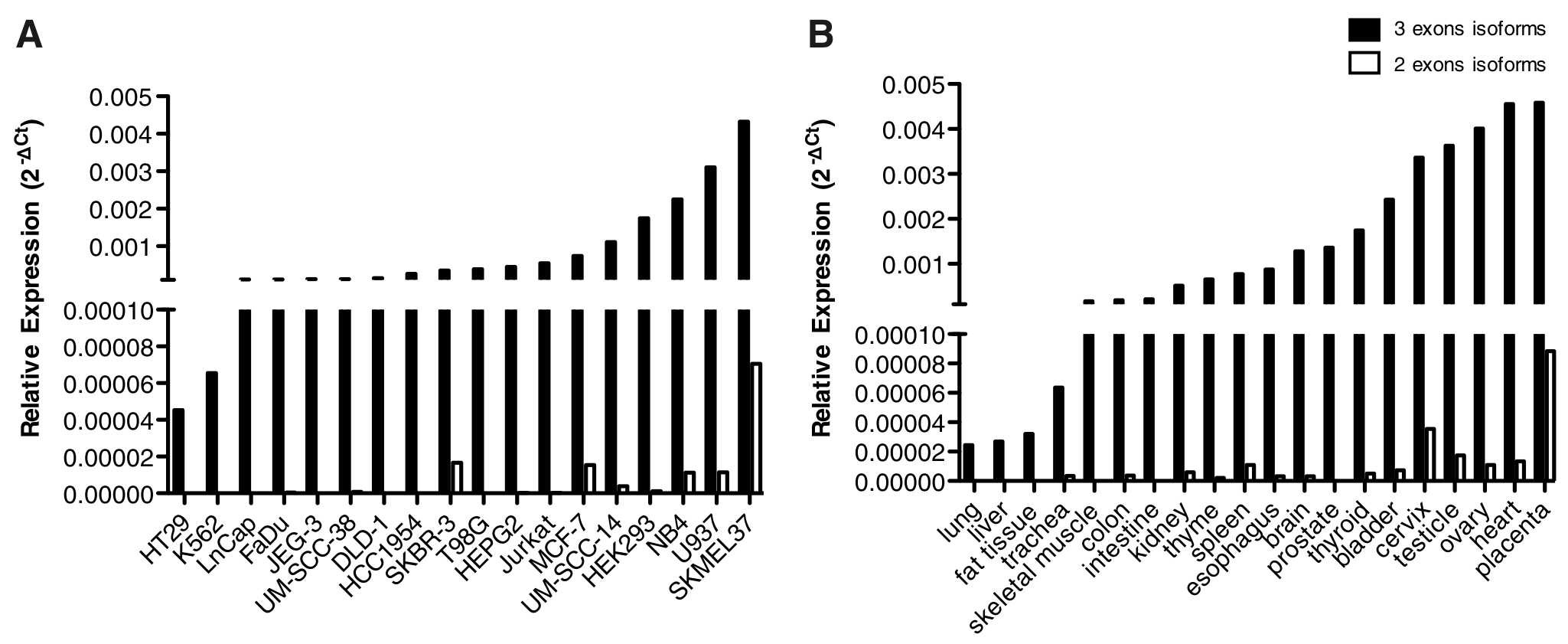

Supplement: S3 Fig — A. LINC00629 isoforms expression pattern in normal tissue panel. B. LINC00629 isoforms expression in cancer cell lines. The endogenous GAPDH and HPRT genes were used for normalization of samples. (TIF) [file pone.0151560.s003.tif]

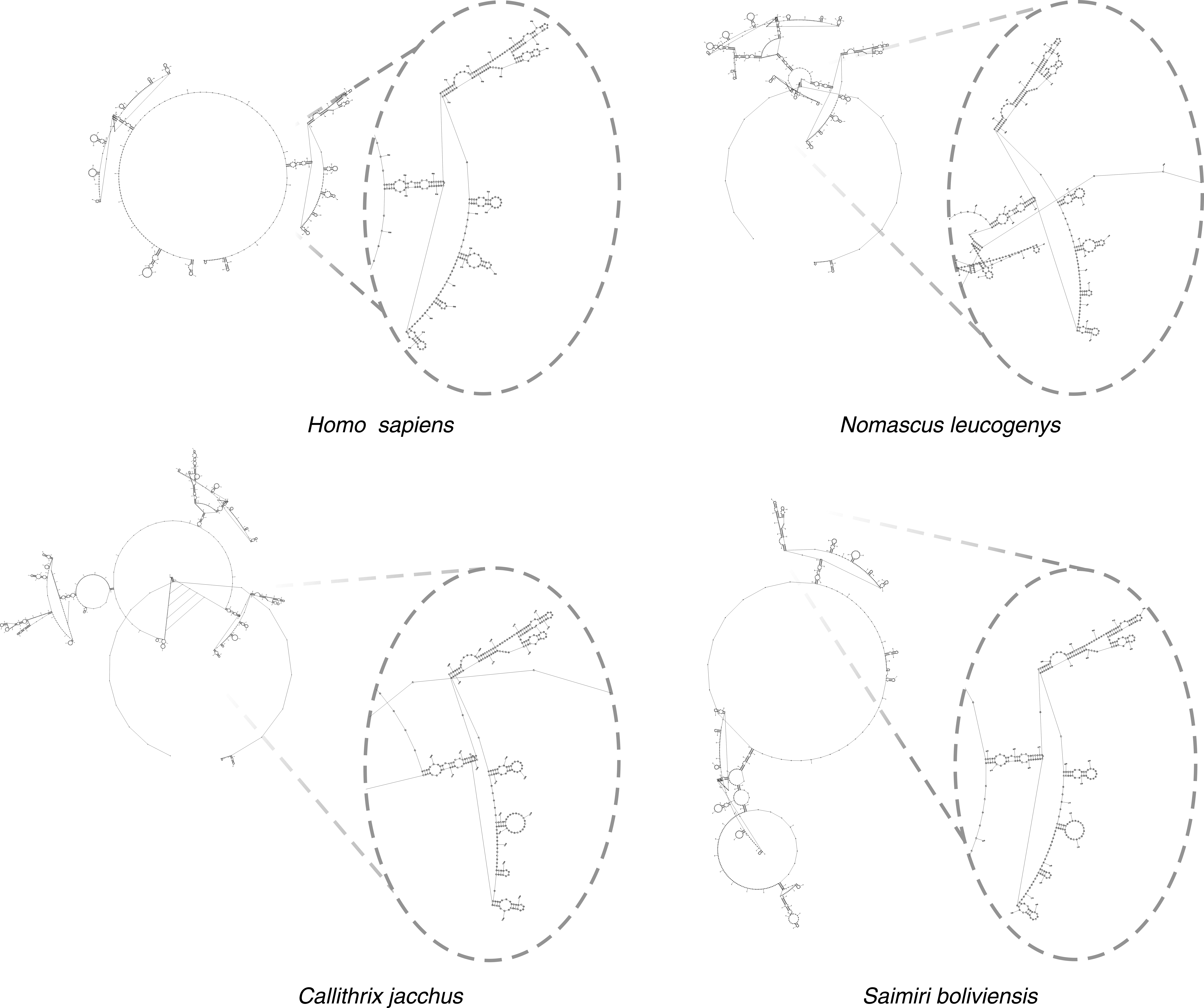

Supplement: S4 Fig — The circulated areas represent the most similar substructure localized in the last exon. The secondary structure was obtained through RNAstructure (http://rna.urmc.rochester.edu/RNAstructureWeb/) using the algorithm TurboFold. (TIF) [file pone.0151560.s004.tif]

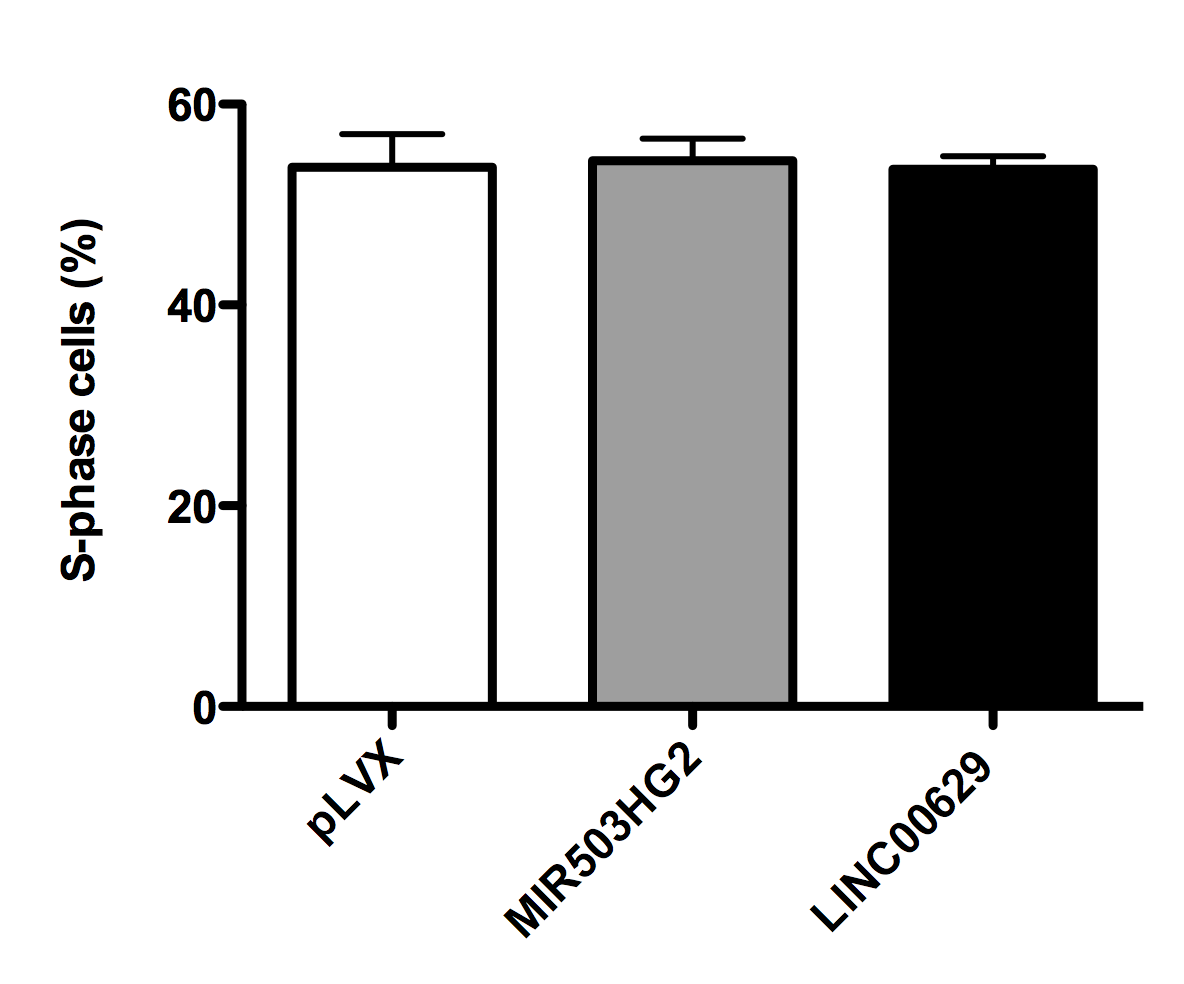

Supplement: S5 Fig — After 48 hours from transfection, cells were fixed with ice-cold absolute ethanol overnight, added to RNAse A and PI and analyzed in FACS Calibur flow cytometer. pLVX: empty expression vector. (TIFF) [file pone.0151560.s005.tiff]
